# Improving Hospital at Home for frail older people: insights from a quality improvement project to achieve change across regional health and social care sectors

**DOI:** 10.1186/s12913-017-2334-9

**Published:** 2017-06-05

**Authors:** M. Pearson, A. Hemsley, R. Blackwell, L. Pegg, L. Custerson

**Affiliations:** 1 Collaboration for Leadership in Applied Health Research and Care (CLAHRC, South West Peninsula, Institute of Health Research, University of Exeter Medical School, St Luke’s Campus, Exeter, EX1 2LU UK; 2 Royal Devon and Exeter NHS Foundation Trust, Barrack Road, Exeter, EX2 5DW UK; 3 South West Academic Health Science Network (SW AHSN), Pynes Hill Court, Pynes Hill, Exeter, EX2 5SP UK; 4 Newton Abbot Hospital, West Golds Road, Jetty Marsh, Newton Abbot, Devon TQ12 2TS UK

**Keywords:** Frail older people, Hospital at home, Quality improvement, Integrated care

## Abstract

**Background:**

Against a background of rising numbers of frail older people, there is a need to improve quality and safety of services whilst containing costs. Improving patient outcomes requires change across hospital and community systems. Our objective was to change practice in order to deliver a Hospital at Home programme (admission avoidance and early supported discharge) for frail older people across a regional commissioning area. The programme, undertaken within the Northern, Eastern & Western Devon Clinical Commissioning Group (CCG) sub-localities of Exeter (population 120,000) and Woodbury, Exmouth and Budleigh Salterton (towns with populations of around 10,000), involved reconfiguration of existing services rather than being a stand-alone intervention.

**Methods:**

Quality Improvement methodology, with hospital and community staff using Plan-Do-Study-Act (PDSA) cycles to implement and test service changes. Outcome measures: 1) Discharge destination; 2) Length of stay; 3) Acute Community Team referrals.

**Results:**

Against a backdrop of intense financial pressures, significant community bed closures, and difficult relations between hospital and community services, outcomes remained stable (discharge destination, length of hospital stay, and number of referrals to the community team).

**Conclusion:**

PDSA cycles enabled stakeholders across acute and community services to be involved, promoted a process of collaborative inquiry and ownership of findings, and improved motivation to act on results and produce change. Practitioners and managers seeking to improve the delivery of complex, cross-cutting services in other areas can learn from the experience of applying Quality Improvement methods reported here.

**Electronic supplementary material:**

The online version of this article (doi:10.1186/s12913-017-2334-9) contains supplementary material, which is available to authorized users.

## Background

The provision of care to older people that is proactive, preventive, person-centred and delivered nearer to the patient’s home is a priority from both a quality and cost perspective [1, 2]. Delivering this care to older people with complex co-morbidities requires integrated working across acute and community healthcare teams and the social care sector. In the United Kingdom (UK) this is a central goal of the NHS Health & Social Care Act [3], but one for which there are many organizational barriers [4].

Non-elective admission for older people can be the default response to a crisis and yet these people may not require the services of an acute hospital provider. Patients over the age of 80 years in hospital have the longest length of stay of other age groups and yet the King’s Fund [5] noted that 42–55% of older in-patients could be managed outside of hospital. Older patients’ inappropriately prolonged hospital admissions may be due to a failure of other services, over-medicalisation of crises and risk-averse behaviour. There is therefore a renewed interest in admissions avoidance schemes and early supported discharge.

It is critical that services across health and social care systems improve quality and safety whilst containing costs. ‘Hospital at Home’ for frail older people, involving admission avoidance (typically proactive health and social care services designed to support people in the community to avoid them ‘tipping’ into needing acute hospital care) and early supported discharge, is a key way of reconfiguring complex services and working relationships in pursuit of this goal.

Hospital at Home targets frail older people, and involves changes in the delivery of hospital and community services. There is - evidence from a systematic review that Hospital at Home programmes that incorporate admission avoidance and early supported discharge can deliver equivalent care to hospital, in an environment that is preferred by older people and their carers [6]. Admission avoidance for older people can reduce mortality rates at six months when compared with standard inpatient care [6] and timely transfer (within two days of the decision to transfer) to early supported discharge can improve functional outcomes for older people [7]. However, understanding of how to implement the system-level changes needed in Hospital at Home remains patchy [8]. Increasing our understanding how Hospital at Home services can be delivered will better enable the delivery of similar evidence-informed system-level changes that improve the quality and safety of services whilst reducing costs.

In Devon the number of people 80 years or older is 20 years ahead of the national curve. Sidmouth, a coastal town in East Devon, currently has the demographics that the UK is projected to have in 2075. Non-acute care for older frail people in the county is oriented towards bed-based intermediate care facilities (often community hospitals) rather than care closer to home, and the extent of integrated care by hospital and community providers is limited. The county’s acute hospital for the north and east (Royal Devon and Exeter) has experienced a 4% year on year rise in non-elective admissions and a non-elective admission rate for patients older than 75 years that is even higher. This situation is unsustainable for the acute hospital provider. The rapid implementation of evidence-based change is therefore a priority.

We sought to create a sustainable comprehensive community based Hospital at Home service for older people in the Exeter sub-locality to enable appropriate admission avoidance and early supported discharge. This required collaborative working between many different organisations and agencies [9, 10]. The new service included a single point of access for referrals, joint geriatrician and community rehabilitation practitioners review for both admission avoidance and early supported discharge, extended weekday and weekend working hours, and an acute hospital ‘step-down’ ward for patients who were no longer acutely unwell. In addition, inter-professional working was facilitated through a focus on joint decision-making, co-location of rehabilitation staff, and an emphasis on implementing changes through consensus. The service was intended to bring about a step-change in the system of care so that community practitioners (amongst others, General Practitioners, Community Nurses, Occupational Therapists, and Physiotherapists) would feel confident and supported to deliver enhanced care in the community, and hospital practitioners would in-turn have sufficient confidence in the system to refer patients for enhanced care in the community. Sponsorship for the project was obtained from senior figures in the acute hospital Trust, community care Trust, and commissioning groups.

Our study focused on the question: What is the impact of using Plan-Do-Study-Act cycles to re-reconfigure and implement a Hospital at Home service for older people in the Exeter sub-locality?

## Methods

The immediate clinical team formed for this improvement project included a Consultant Geriatrician (Lead for Healthcare for Older People at the acute hospital provider) and Senior Occupational Therapist (Lead for the ‘Rapid Assessment at Home Team’ (RAAH) for the community provider). The RAAH team is a multi-disciplinary team consisting of nursing and therapy staff. The extended team included a Data and Performance Manager and Service Manager from the acute trust and an Implementation Science academic from the local University.

The backdrop to the Improvement project was one of significant pressure on resources and strained working relationships between hospital and community teams. Sources of tension included the perception amongst community practitioners that the hospital prioritised discharging patients over their near-term care needs in the community, and a concern that service configuration changes would intensify an already-pressured community workload. Amongst hospital clinicians and practitioners there was a perception that community staff did not appreciate the pressures experienced by acute care staff in providing safe and effective care for non-acutely unwell patients in addition to their core responsibility for acutely unwell patients. Within the sub-locality, both an intermediate rehabilitation facility and 20 beds within the community hospital had been closed. Whilst there was agreement between hospital and community services about the need to safely and appropriately reduce hospital admissions for older frail people, on the frontline there was suspicion about the way that any service reconfiguration would impact on workload, work scheduling, and expectations about responsibilities.

We used an Interrupted Time Series study design with data sourced from the Royal Devon and Exeter NHS Foundation Trust Patient Administration System (PAS). The PAS is the source of the Hospital Episode Statistics [11] which are used to calculate hospital payments. PAS is a widely-used and trusted data source for analysing hospital activity. The structure of the data is defined by the NHS Data Dictionary, with data quality assured through an annual Payment by Results Data Assurance Framework Audit undertaken by the Audit Commission. The referral data was sourced from the Acute Community Service own data collection which is not subject to any data quality assurance checks.

Through discussion and critical reflection on practice within the project team, we developed a driver diagram to show how we envisaged service configuration in four areas - assessment processes, support services, resources, and integrated working. Action was necessary at a number of levels within each of these areas. For example, assessment required changes at the levels of: 1) individual practitioners (understanding revised assessment processes); 2) team leaders (building shared strategic goals); 3) system processes (shared documentation and referral processes); and 4) financial resources (to support transition).

We used Plan-do-study-act (PDSA) cycles as an established Quality Improvement (QI) tool. The four stage framework facilitates ‘iterative testing’ in complex systems, allowing interventions to be undertaken on a small scale with rapid evaluation. This can then inform further interventions or tests of change. A recent systematic review identified benefits of using a QI approach, including flexibility, freedom of approach, engagement and evidence building [12].

We used PDSA cycles throughout the improvement project. These were undertaken at all stages of the patient pathway and varied in scale, focus and anticipated impact. The majority of the PDSA cycles were undertaken between the Consultant Geriatrician and the RAAH team. The PDSA cycles took place both within the acute hospital (for example, joint review and discharge plan of a patient with complex care needs) and the community (for example, Consultant Geriatrician input to the rapid review of a patient for admission avoidance). PDSA cycles were also used to test alternative ways of working with partner organisations, such as the local mental health trust. This enabled both small scale changes with a single patient or team member and large scale interventions (for example, extended weekday hours, or weekend working) to be tested. Emerging findings from the PDSA cycles fed back into the multi-faceted service reconfiguration process - for example, as evidence to support the case for financial or human resources to support implementation. A themed summary of the PDSA cycles, including the learning from each, is shown in Additional file 1.

As our study used routinely-collected data and was conducted under the auspices of a nationally-recognised Quality Improvement programme, ethical approval was not required.

### Study design

Interrupted time series, using Hospital Episode Statistics on discharge destination, length of hospital stay, and Acute Community Team referrals for all patients in the period April 2011 to March 2014 (*n* = 8055) aged 80 years or over who were acute medical admissions to the city hospital and who were registered with a General Practitioner in the city. Statistical Process Control (SPC) charts were initially used to identify any special cause variation present during the project. The P Chart is used to plot percentages of conforming (or non-conforming) units per subgroup where the subgroup size may be constant or varying and was used to plot the percentage of Exeter GP Registered Patients aged 80 or over discharged to their usual place of residence from the Royal Devon and Exeter where the subgroup was the number of discharges in that week. The Xbar chart is used to plot the mean of a subgroup where the subgroup size varies and was used to plot the mean length of stay of Exeter GP Registered Patients aged 80 or over discharged from the Royal Devon and Exeter where the subgroup was the number of discharges in that week. The X Chart (or I Chart) is used to plot individual values and was used to plot the number of pull referrals from the Royal Devon and Exeter accepted by the community team.

The SPC methodology used was as set out in the Health Care Data Guide [13] and is used to distinguish between ‘normal cause’ and ‘special cause’ variation. This allows for the identification of patterns in the data that are not part of the normal variation or noise in the system. For example a data point lying outside the 3 sigma control limits is unlikely to be part of the ‘normal cause’ variation and is more likely to be the result of some external stimuli and should this be present at the time of an experimental intervention and the absence of any other known stimuli it would suggest that the intervention was responsible for this ‘special cause’ variation. See Additional file 2 for the analyses using SPC charts.

In view of the risk of insufficient sensitivity to change in the SPC analyses, we subsequently analysed discharge and length of stay data using Cumulative Sum (CUSUM). We also transformed the Acute Community Team referrals data in view of its non-normality prior to calculating the control limits for the X chart. The transformation function used for this was the double square root:

$$ f(x)=\sqrt{\sqrt{x}}={x}^{\frac{1}{4}} $$

The resulting transformed data was then used to create the control limits as per normal X Chart construction and then the values for the control limits were re-transformed using the inverse of the transform function (to create the chart shown in Fig. 3)

$$ f(x)={\left({x}^2\right)}^2={x}^4 $$

## Results

Findings from the 12-month QI project underpinned service reconfiguration. The RAAH team expanded in scope and scale, developing from an admission avoidance service (not integrated with acute hospital care) to an admission avoidance and early supported discharge service that worked closely with the acute hospital. The RAAH team expanded from six to fourteen members of staff, including a Clinical Nurse Specialist and Community Psychiatric Nurse. Changes in the provision of acute hospital beds and the structure of community services were co-ordinated to achieve more care in the community, with working relationships and understanding between acute and community care considerably improved, and changes in community working patterns achieved to provide seven-day care.

The percentage of patients registered with a GP practice in the Exeter locality who were discharged to their usual place of residence is shown in Additional file 2. By recalculating the limits from the start of the special cause variation, it can be seen that there are no signs of special cause variation following the rebasing. This indicates that the process remains stable at the new level, with the percentage of Exeter patients being discharged back to their usual place of residence having increased from 75.85% to 80.99%. The proportion of patients being discharged to Exeter Community Hospital remained stable (data not shown).

**Fig. 1:**
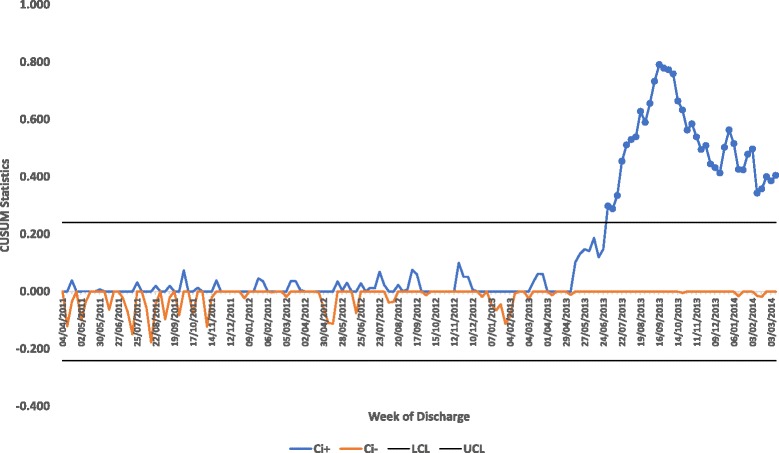
Rebased P Chart of Exeter GP Registered Patients Aged 80 and over Discharged to their Usual Place of Residence

The percentage of Exeter GP registered patients aged 80 and over discharged to their usual place of residence from the Royal Devon and Exeter is shown in the CUSUM chart in Fig. 1. This shows that the positive CUSUM statistic line exceeds the upper control limit from the week beginning 01/07/2013 which approximates the analysis shown in the SPC chart (rebased from the last week in June 2013, see Additional file 2).

The length of stay for patients registered with an Exeter GP is shown in Additional file 2. There was special cause variation (8 or more points below the centreline) during the periods 01/10/2012 and 03/12/2012 and a further outlying point on the 06/01/2014 (which was likely to have been caused by limited access to community services over the Christmas period).

**Fig. 2:**
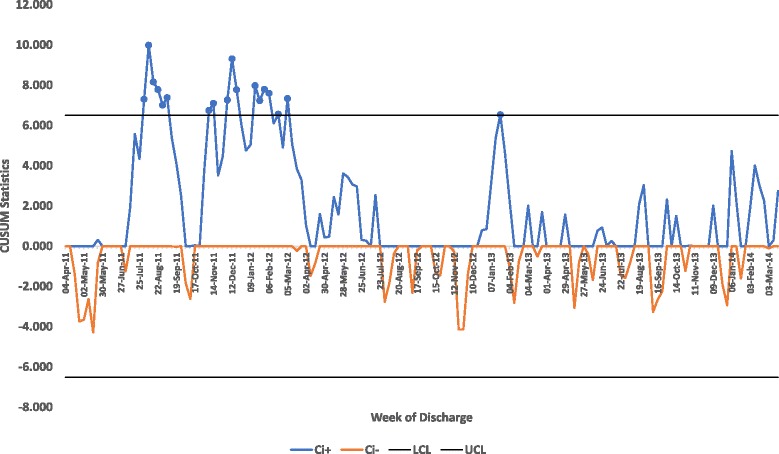
X bar Chart of Length of Stay of Exeter GP Registered Patients Discharged from the Royal Devon and Exeter

The mean length of stay Exeter GP registered patients aged 80 and over discharged during that week from the Royal Devon and Exeter is shown in the CUSUM chart in Fig. 2. For the periods July 2011 to beginning of September 2011, November 2011 to beginning of March 2012 and the week beginning 21/01/2013 where the positive CUSUM statistic exceeded the upper control limit which would indicate on these occasions there had been an increase in the length of stay. This is not apparent in the XBar SPC chart where the only triggers are for the period 01/10/2012 and 03/12/2012 (8 or more points under the centreline) and one outlying point on the 06/01/2014.

The X Chart of the number of push referrals accepted by the Acute Community Team shows no special cause variation, with an average of 25 push referrals per month accepted (data not shown). The X Chart of pull referrals accepted by the Acute Community Team transformed using a double square root transformation prior to adjusting for non-normality in the data (see Additional file 2) shows that from July 2013 to March 2014 there were 9 consecutive points above the mean indicating special cause variation and a significant increase in the number of referrals accepted, as can be seen in the last five data points.

**Fig. 3:**
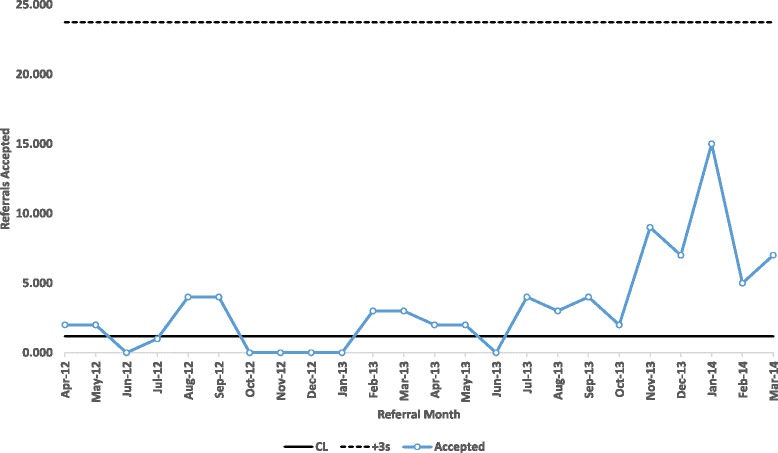
X Chart of Royal Devon and Exeter (Pull) Referrals Accepted by the Acute Community Team

The number of pull referrals accepted by the acute community team is shown in the CUSUM chart in Fig. 3. This shows that the negative CUSUM statistic triggers the lower control limit between November 2012 and November 2013 which would indicate that the number of referrals accepted had fallen and the positive CUSUM statistic triggers the upper control limit from Dec-13 onwards indicating that the number of referrals accepted for that period had increased.

## Discussion

At face value, the outcomes of our QI project appear unremarkable. Discharge destination and length of hospital stay remained stable, as did the number of referrals to the community team. However, such an analysis ignores the wider context of intense financial pressures within the NHS and significant community bed closures (leading to an intensification of complex cases for community care). There was also a local history of difficult relations between hospital and community services. Service reconfigurations that maintain stability against such a backdrop and which lead to important signals of improvement are a success. They also provide the bedrock for future improvement in the quality, safety and efficiency of integrated hospital and community services, as well as between adult social care, mental and physical health care, and acute and long-term services. In our locality, an upward trajectory in integrated working is suggested by the seven-fold increase in pro-active ‘pull’ referrals from the community and a step-change in the quality of working relationships between hospital and community staff.

Our use of PDSA cycles demonstrated the significance of working collaboratively across teams. Building productive working relationships between teams required recognising strengths and weaknesses and working together to reconfigure services in a way that was patient-centred. Supportive relationships across teams, united by the strategic vision provided by Geriatrician involvement, provided the framework on which referral and information-sharing processes could be built. These supportive relationships also enabled practitioners to feel more secure in moving services towards a pro-active, patient-centred, therapy-led approach.

There are important limitations that we acknowledge in relation to this QI project. We did not collect data about other aspects of care quality (such as patient experience or shared decision-making), case-mix, or other variables (such as General Practitioner sickness) which may have impacted on the findings. Conducting the project within our day-to-day service roles meant that we did not have the resources to make comparisons with Hospital at Home projects in other regions. Our inferences about PDSA cycles providing a mechanism for increasing understanding within and between hospital and community teams are based on observation and reflection rather than being established by testing. More explicit testing of the precise ways in which PDSA cycles facilitate changes in practice is required.

Achieving change within a complex system is a long-term project. We recognise that there were methodological limitations in our QI project, but are also mindful that using QI methodology enabled us to build and maintain constructive working relationships that were supported by organisational changes to facilitate integrated working. PDSA cycles aided the joint identification of problems and provided a mechanism for discussing and testing potential solutions that involved all members of the hospital and community teams. The process, including the feedback of findings from the PDSA cycles, built ‘ownership’ of service changes and the motivation to continue to deliver them.

## Conclusions

Quality Improvement can be a useful methodology in health and social service development across the primary and secondary care boundary. We found that rapid cycles of PDSA tests facilitated iterative learning that in turn enabled service change to occur at pace. The way in which PDSA tests enabled stakeholders across acute and community services to be involved promoted a process of collaborative inquiry. This in turn enabled the development of a deeper awareness and understanding of the issues, greater ownership of the findings and ultimately a heightened motivation to act on the results and produce change. However, more explicit testing of the ways in which this process of change occurs is needed. Contextual differences in service history means that whilst the components of the service reconfiguration used in this project cannot be considered a template, practitioners and managers seeking to improve the delivery of complex, cross-cutting services in other areas can nevertheless learn from the experience of applying Quality Improvement methods reported here.

## Additional files

Additional file 1: PDSA cycles summary. (DOCX 20 kb)

Additional file 2: SPC Charts. (DOCX 525 kb)

## Abbreviations

NHS: National Health Service
PAS: Patient administration system
PbR: Payment by Results
PDSA: Plan-do-study-act cycle
QI: Quality improvement
RAAH: Rapid assessment at home team
SPC: Statistical process control
