# Supplementary material for: Improving Hospital at Home for frail older people: insights from a quality improvement project to achieve change across regional health and social care sectors
Source: BMC Health Serv Res. 2017 Jun 5;17:387. doi: 10.1186/s12913-017-2334-9 (PMC5460362; doi:10.1186/s12913-017-2334-9)
Supplement: Supplementary file 1 — PDSA cycles summary. (DOCX 20 kb) [file 12913_2017_2334_MOESM1_ESM.docx]

| **PDSA subject** | **Action** | **Outcome** | **Learning** | **Date/PDSA #** |
| --- | --- | --- | --- | --- |
| Approach to collaborative working between the acute and community setting | Engagement with RAAH team to jointly work with RD&E towards a shared vision for admission avoidance and early supported discharge for frail elderly in Exeter  Discussion between services and understanding of different approaches / needs and demands  Joint assessment with team lead and consultant to build up rapport, communication and trust and to enable a better understanding of the patient journey between the community and acute trust. | Improved engagement and trust between the acute setting and community.  Discussion around how they can support each other to ensure the best outcome for the patient. Learn from each other’s experiences, knowledge and ideas. | Trust and excellent communication is needed to establish collaborative working. | 1.a: 04/04/13  1b: 26/11/13 |
| Collaborative / joint working between different organisations | Social services; patients presenting with acute illness who have a social care need outside of the RAAH skill base and will need a social care assessment for the appropriate intervention such as urgent respite care.  Community Rehab Team; following an acute episode the patient may need a period of rehabilitation which may go on beyond our agreed level of service delivery. A joint visit will support change over and where as a band 6 would be required to compete an initial assessment, when transfer form RAAH band 6 to a ECH band 4 / 3 reduces impact on band 6 in ECH and enhances the change over from RAAH to ECR  Mental health services; patients presenting with an acute illness and also have a primary mental health diagnosis affecting their function therefore requires the skills of both an acute care services and mental health services for appropriate and safe intervention.  There is also evidence of differences in each services understanding of each others services, jargon, intervention and culture. Joint working helps understanding and eases frustrations between services, policies and procedures. Also able to delegate out appropriate intervention between both services to ensure the patients gets the best care delivery and intervention. | Patients can be passed to and fro between services leaving patients at high risk. RAAH can support patients but on-going needs need to be addressed by other services.  RAAH can highlight needs, prevent / diffuse the crisis but the basic needs remain. PDSA has evidenced the benefit of joint assessment between services to streamline assessments, delays between services are reduced and the patients has a more seamless intervention.  Valuable learning between each team and how they assess and work. Increased learning and understanding supports communication and intervention.  Built stronger relationships with community rehabilitation services and developed an improved referral process to save duplication of assessment and ease transfer of patient.  We have gained funding and recruited a community care worker in the team to support with social care needs.  We are looking for a simplified pathway to access mental health services to support ACT patients when indicated. | Collaborative working with services highlighted the need for all services to respond towards the patients’ needs and be responsive.  Joint working helps to educate services of each other’s need and roles and importance to support each other to get the best for the patients.  Referral process to MH services is via GP and not direct to their own services which limits access to gain support from our mental health colleagues. The criteria restricts access as they are reluctant to assess patients who are acutely unwell who may be toxic, stating this will affect their assessment. But denies patient’s form a skilled assessment form mental health services – collaborative working would be of benefit and joint assessments etc. | 2.a:  08/01/14  2.b: 24/07/13 |
| Changes in service delivery | Recup Beds, urgent care beds for patients requiring 24 hour care but not acute care.  24 hour care at home  Changes to support avoidable admissions and incorporate supported discharges  Changes to patient admission for diagnostics – ACT and ACE team working collaboratively to improve patients’ journey and diagnostics. | Helped to highlight the need for a better service for recuperative care and access to urgent care beds (non-acute).  Regular PDSA helps to address needs and evidence the need for change. | It has helped to get people and others services to think about this service need differently.  This is an on-going issue and we continue to work towards accessing improved recuperative care system and block purchase beds. | 3.a: 1/5/13  3.b: 5/6/13 |
| Addressing issues in joint management meetings (both Acute and community settings) | Closer working between primary and secondary care setting in Exeter  Working towards geriatrician involvement with RAAH  Inappropriate use of ACT and impact this has on the team, service provision and capacity  Waiting lists for community hospital have decreased as we are able to support more patients to remain at home safely and reducing impact on primary and secondary care settings and we also support discharges from the secondary care setting avoiding the need of a community hospital bed. Rehabilitation in their own home tends to increase their engagement their rehabilitation programme as they motivated in their own environment (loss of community beds in 2013 due to ward shutting also impacted on increase in patient accessing the service as no beds available in community hospital – more complex patients tended to get referred now).  Highlight gaps in service once ACT are ready to discharge patients.  Partly due to waiting lists and referral processes and protocols. | Aided in understanding of cultural and role differences.  Communication became easier as we learnt to understand the jargon and terminology of each other and realised we had the same vision but communicated in different ways.  Support from having a geriatrician highlighted the service development in the RD&E, offered medical support to team members and allowed easier access for our patient who caused concern to be able to access either a verbal or face to face consultation quickly and seamlessly.  Patients were supported and confident in the service as their wishes to remain at home were respected. Staff felt safer with service delivery and supported medically.  Pathways and process improved between community and acute trust services.  Support was gained for approaching change and developing vision between primary and secondary care. | Highlighted the need for geriatrician / GP involvement as ACT do not currently have this in place. Able to show how geriatrician involvement aided patient journey and treatment in both the community and acute setting.  Education and training of services and how we can work collaboratively to ensure the patient gets the best experience, right care at the right time in the right place | 5.a: 24/07/13  5.b: 24/09/13  5.c: 24/09/13 |
| Patient Care | There has been an increase in the number of complex patients being discharged home due to a shortage of beds. Skills in the team are challenged as we provide a service which aims to support these patients to remain at home safely. This requires an increase in RAAH service assessments, increase in equipment provision, increased demand on other services to provide care daily and night sits. Due to the complexity of the patients and their situation a multidisciplinary approach is required, being flexible to meet the demands on the service. Complex patients and involvement required from a multidisciplinary team and time constraints | Re-design of RAAH team  Evidence need for increase in staffing  Different approach to support patients in the community as medical and social care model is not sufficient.  Ned to highlight and address the impact on other services such as rapid response care provision and there had been an increase in recruitment to support demand.  Increase in demand on training to support staff in caring for more acutely patients to remain at home safely – highlighted to senior management  Highlighted need for equipment provision at weekend  Need of a private emergency equipment cupboard to acute need  Service provision was initially set at intervention for 6 weeks. It was reduced to 5 days. This challenged the service as most acute illnesses take 3 -5 days to see an improvement. Therefore no time to rehab to previous level of function. RAAH could not complete rehab so patients were referred on to community rehab team and increased their waiting list and impacted on their service. We managed to get it increased to 10 days as that is the average length of stay, and then eventually to 21 days. | Able to highlight the impact some more complex patents have on a small team. Whilst they have the skill, the requirements and demands on the team affect capacity.  It also highlights the need for other services to work extended hours and weekends to support ACT to ensure quality service patients risk and improve support to remain at home safely.  Evidence the need for acute community services to be involved with acutely unwell patients for 21 days as 5 days is not enough due to patients needing to be medically stable and feel well enough to engage with therapy to support their rehabilitation to get them back to their normal level of function before discharge. Allows for continuity of care and reduced the patient need for duplication of information between services when referred on too soon. | 6.a:02/03/15 |
