# Supplementary figures and images for: Improving Hospital at Home for frail older people: insights from a quality improvement project to achieve change across regional health and social care sectors
Source: BMC Health Serv Res. 2017 Jun 5;17:387. doi: 10.1186/s12913-017-2334-9 (PMC5460362; doi:10.1186/s12913-017-2334-9)

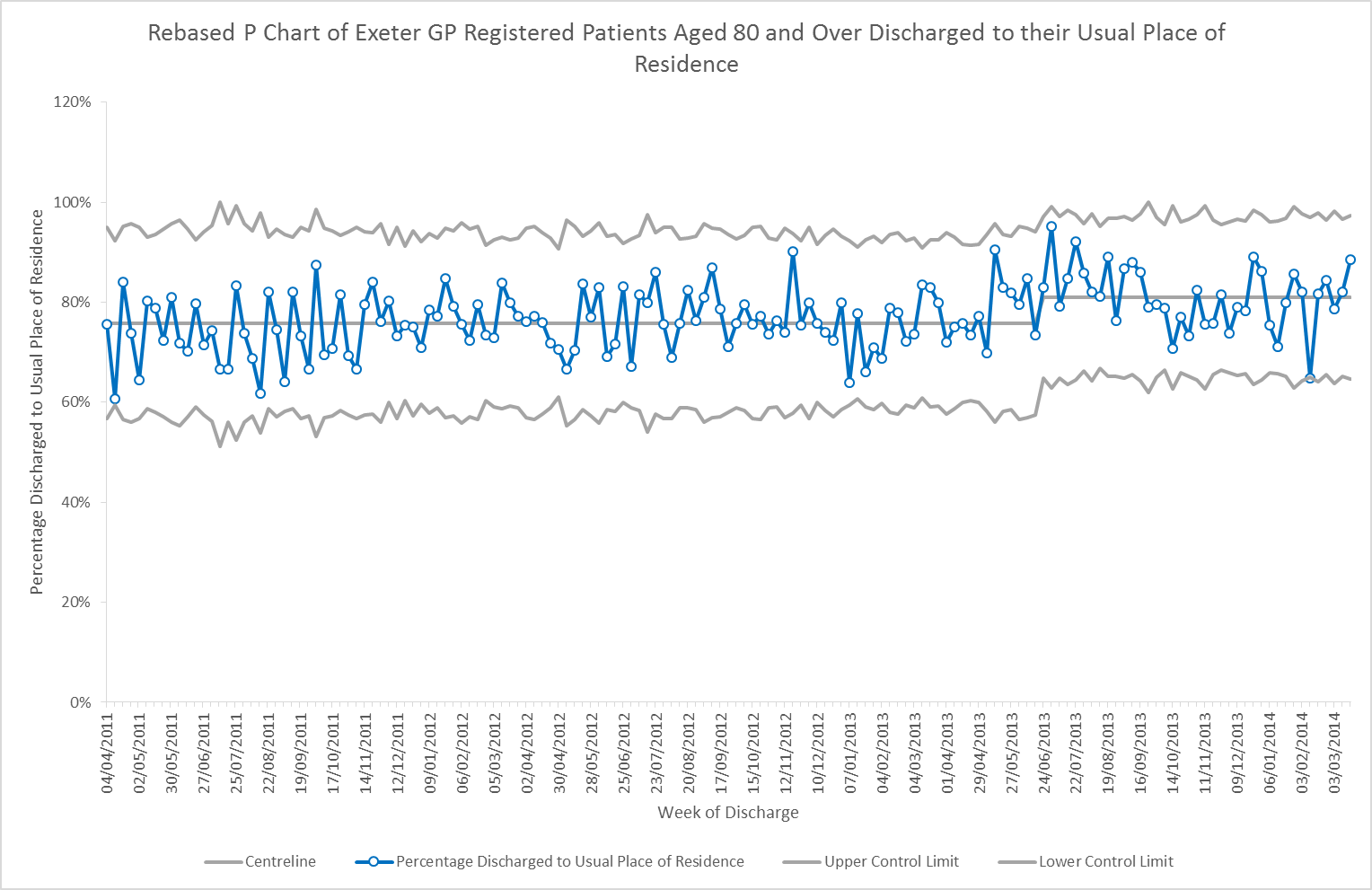

Supplement: Supplementary file 2 — SPC Charts. (DOCX 525 kb) [file 12913_2017_2334_MOESM2_ESM.docx]
